# Quercetin Alleviates Ferroptosis of Pancreatic β Cells in Type 2 Diabetes

**DOI:** 10.3390/nu12102954

**Published:** 2020-09-27

**Authors:** Dan Li, Chunjie Jiang, Guibin Mei, Ying Zhao, Li Chen, Jingjing Liu, Yuhan Tang, Chao Gao, Ping Yao

**Affiliations:** 1 Department of Nutrition and Food Hygiene, School of Public Health, Tongji Medical College, Huazhong University of Science and Technology, Wuhan 430000, China; lidangoodgirl@163.com (D.L.); jcj2010150044@163.com (C.J.); 15695903945@163.com (G.M.); stuzhaoying@163.com (Y.Z.); cl707339221@163.com (L.C.); liujingjing690@163.com (J.L.); tyh043@126.com (Y.T.); 2 National Institute for Nutrition and Health, Chinese Center for Disease Control and Prevention, Beijing 100000, China; gaochao20090901@163.com; 3 Ministry of Education Key Laboratory of Environment School of Public Health, Tongji Medical College, Huazhong University of Science and Technology, Wuhan 430000, China

**Keywords:** ferroptosis, quercetin, type 2 diabetes mellitus, iron overload, lipid peroxidation

## Abstract

(1) Background: Pancreatic iron deposition has been found in the progression of type 2 diabetes (T2DM); however, whether ferroptosis contributes to the dysfunction of pancreatic β cells (PBC) remains enigmatic. Moreover, the potential protective effect of quercetin is also elusive; (2) Methods: T2DM mice model was established by multiple low dose streptozocin (STZ) injection, after which quercetin was intervened for 4 months; (3) Results: Substantially normalized glucose tolerance, diabetic symptoms, homeostasis model assessment for insulin resistance (HOMA-IR), and homeostasis model assessment for β cell (HOMA-β) index in comparison with the findings of T2DM control. Distorted pancreatic islets and especially shrunken mitochondria with cristae loss in PBC were observed in T2DM mice, which was ameliorated by quercetin. Meanwhile, quercetin lowered the iron level particularly in the islet in T2DM mice. In spite of compensatory xCT up-regulation, T2DM molding depleted glutathione (GSH), down-regulated glutathione peroxidase 4 (GPX4), and induced oxidative stress in pancreatic tissue, which was abolished partially by quercetin. More importantly, insulin secretion was worsened by ferroptosis-inducing erastin or RAS-selective lethal compounds 3 (RSL-3). Quercetin, ferroptosis inhibitor ferrostatin-1 and iron-chelating deferoxamine, rescued cell viability when cells were challenged with high-glucose; (4) Conclusions: Our findings identify that ferroptosis contributes to the PBC loss and dysfunction. Quercetin exerts beneficial effects on T2DM potentially by inhibiting pancreatic iron deposition and PBC ferroptosis, highlighting promising control strategies of T2DM by quercetin.

## 1. Introduction

Diabetes mellitus (DM) is one of the fastest-growing global health emergencies. The latest estimate from the International Diabetes Federation comes to 463 million (9.3% of the global population) in 2019, an alarmingly tripling increase since 2000. The number would jump to 578 million (10.2%) by 2030, and a staggering 700 million (10.9%) by 2045 [1]. Type 2 diabetes mellitus (T2DM) accounts for the vast majority (around 90%) of DM. Individuals with T2DM are at high risk for both microvascular complications (including retinopathy, nephropathy, and neuropathy) and macrovascular complications (such as cardiovascular comorbidities) owing to hyperglycemia and insulin resistance (IR) syndrome [2], imposing a heavy burden on economic and health systems in the world.

Apart from well-studied IR in peripheral cells, impaired insulin secretion has been acknowledged as the core defect of severe T2DM because of the decompensation of pancreatic β cells (PBC) after long-term glucotoxicity and lipotoxicity [3,4]. Responsible for impoverished insulin synthesis and secretion, the shrunk pancreatic islet and reduced PBC mass are frequently reported on T2DM biopsy [5], which attracts extensive attention to decipher the underlying molecular mechanisms behind the phenotypes. PBC death involving the modulation of PBC mass and function has been presumed as a final event and critical strike on the progression of T2DM, leading to rapid deterioration of various complications [6]. Nevertheless, the precise mechanisms of PBC death still largely remain elusive in spite of multitudinous efforts, especially on the apoptosis and necrosis [7,8,9].

Iron is an important nutrient with extensive physiological function evidenced by iron deficiency; however, iron overload has been found to link with various human diseases, including cancer, atherosclerosis, etc. [10,11]. For T2DM, abnormal iron status has been observed in a large number of cross-sectional surveys [12], and further verified by emerging cohort studies [13]. Clinical findings showed an excessive iron deposition in the pancreas, and particularly in pancreatic islet, when suffered from T2DM [14]. Functioning as a vigorous catalyst of Fenton reaction, it is plausible that pancreatic iron deposition may contribute to the loss or death of PBC.

Ferroptosis, a newly-identified form of regulated cell death, characterized by irreparable lipid peroxidation resulted from over-generation of reactive oxygen species (ROS) in an iron-dependent manner, has demonstrated to play a fundamentally pathological role in various diseases associated with iron overload or dysfunction [15,16]. Since the discovery of ferroptosis, other types of cell death have gained extra concern for the same features as ferroptosis, despite morphological and biochemical distinction and disparition. Morphologically, ferroptosis is characterized by decreased mitochondrial volume, increased mitochondrial membrane density, decreased or disappeared mitochondrial cristae, rupture of mitochondrial outer membrane, while normal nucleus size remains, nucleation-free shrinkage or chromatin condensation, which are the most important differences from apoptosis and necrosis. Biochemically, while apoptosis is triggered by the release of intermembrane space (IMS) proteins and is dependent on caspases, and necrosis is characterized nicotinamide adenine dinucleotide phosphate(NADPH) oxidase activation and neutrophil extracellular trap (NETs) release from the neutrophil trap. In ferroptosis, most important features of ferroptosis are lipid peroxides (LOOH) and increased ferrous ions (Fe^2+^) concentration. Overproduction of ROS, which is produced by Fenton reaction will cause lipid peroxidation in cells. To our knowledge, little attention has focused on the PBC ferroptosis implicated in T2DM pathogenesis, even if the findings are common on pancreatic oxidative stress and fragile antioxidant capacity of PBC [17].

Quercetin (3,3′,4′,5,7-pentahydroxyflavone), as one of the most widely distributed flavonoids in the plant kingdom, has been reported a mass of attractive physiological function including reducing the risk of T2DM in the epidemiological investigation [18]. Such potentially anti-diabetic effect is observed on laboratory research in vivo and in vitro, several underlying mechanisms have been proposed, including the stimulation of insulin secretion [19], anti-oxidative and anti-inflammatory protection on pancreas or PBC [20], IR attenuation of peripheral tissues [21], and so on. Serving as a naturally occurring inhibitor or regulator of iron metabolism, quercetin is beneficial on iron overload related diseases. Furthermore, recent studies showed that polyphenols, including quercetin, curcumin, and epigallocatechin-3-gallate may present as a potent inhibitor of ferroptosis but not apoptosis [22,23]. However, up to now, the potential anti-diabetic role of quercetin on PBC ferroptosis has not been explored. Herein, we designed the present study to further investigate the mechanisms by which quercetin protects from T2DM, aiming to shed new light on T2DM prevention by natural phytochemicals.

## 2. Materials and Methods

### 2.1. Chemicals and Reragents

Quercetin (purity ≥98%, HPLC) and streptozocin (STZ) were purchased from Sigma-Aldrich (St Louis, MO USA). Anti-xCT rabbit monoclonal antibody (ab175186), anti-GPX4 rabbit monoclonal antibody(ab125066), anti-VDAC2 goat polyclonal antibody (ab37985), anti-Ferritin light chain (ab69090) were purchased from Abcam (Cambridge, UK), and anti-β-actin mouse monoclonal antibody (3700s), and secondary antibody anti-rabbit IgG, anti-mouse IgG, and goat anti-rabbit IgG were obtained from cell signaling technology (Danvers, MA, USA), anti-insulin antibody was brought from Santa Cruz Biotechnology (sc-8033, Santa Cruz, CA, USA). Serum levels of insulin were estimated using the Mercodia insulin ELISA kits (Mercodia, Uppsala, Sweden), whereas insulin level in cellular supernatant was determined by radioimmunoprecipitation (Beijing BeiFang Biotech, Beijing, China), and serum iron level was detected by serum iron assay kit (Nanjing Jiancheng, Nanjing, China) according to the manufacturer’s instructions. glutathione (GSH), superoxide dismutase (SOD) activity, and lipid peroxide in tissue were quantified by GSH kit (Nanjing Jiancheng, Nanjing, China), SOD kit (Beyotime, Haimen, China), malondialdehyde (MDA) kit (Beyotime, Haimen, China), respectively. Lipid peroxidation in INS-1cell was accessed with C11-bodipy (Thermo Fisher Scientific, Waltham, MA, USA), ferrostatin-1 (2 μM), necrostain-1(10 μM), Z-VAD-FMK (10 μM), was purchased from Med Chem Express Corporation (Monmouth Junction, NJ, USA). Other chemicals and reagents were of analytic grade.

### 2.2. Animal Study Design

Male specific-pathogen-free C57BL/6J mice, aged 7–8 weeks, weighing 19–21 g, were purchased from Beijing Vital River Laboratory Animal Technology Co., Ltd. (Shanghai, China). The experiement was started after acclimation to laboratory conditions for 1 week. Sixty mice were randomized into 2 groups with 30 each, fed by a high-fat diet (HFD, 60% fat) or low-fat diet (LFD, 10% fat) for 4 months, respectively. Streptozotocin (STZ) was then intraperitoneally injected to HFD-fed mice (for 5 consecutive days, 50 mg/kg body weight; dissolved in citrate buffer 0.1 M, pH 4.5) to further induce T2DM model [24]. Animals with blood glucose levels over 16.7 mmol/L measured by Accu-Chek Performa glucometer (Roche Diagnostics, Mannheim, Germany) 3 days after STZ injection were considered diabetic [21]. Subsequently, all diabetic mice and the LFD-fed mice were randomly divided into 2 subgroups as follows: (1) normal control (C, LFD), (2) quercetin control (Q, LFD+Q), (3) T2DM model (DM, HFD), (4) quercetin intervention (DM+Q, HFD+Q). Quercetin (1.5 g/kg) was administrated for another 4 months, and quercetin is intervened with feed blending, so that animals have free access to food. The average applied dosage is 100 mg/kg·body weight. All animals were maintained under standardized conditions of good ventilation, room temperature 23 ± 2 °C, relative humidity 55 ± 5%, and 12 h light/dark cycle. Animals were provided with a corresponding diet and allowed free access to tap water. Bodyweight was measured weekly and food and water consumption were calculated daily. At the end of the experiments, blood was taken from the orbit of mice after anesthesia by chloral hydrate (6%) with intraperitoneal injection (0.05 mL/10 g) and serum was separated and then stored at −80 °C for the biochemical estimation, the pancreas was dissected, weighed, and stored at −80 °C for further analysis. All experiments performed were approved by the Tongji Medical College Council on Animals Care Committee. The code is [2017] S820, and the project identification code is No.81602858.

### 2.3. Oral Glucose Tolerance Test and Insulin Tolerance Test

Food was withheld 8 h before testing. Mice were administrated with glucose (2 g/kg) by oral gavage. Small blood samples (typically 5 μL) were taken by tail prick at 0, 15, 30, 45, 60, 90, and 120 min relative to glucose administration for the measurement of blood glucose levels using Roche glucometer. Additionally, when the insulin tolerance test was carried out, 0.75 IU/kg insulin was injected intraperitoneally after basic blood glucose levels were determined. The measurement of blood glucose was the same as oral glucose tolerance.

### 2.4. Determination of the Biochemical Parameters

Serum levels of glucose and iron were estimated using the corresponding colorimetric kit according to the manufacturer’s instructions. Mouse-specific ELISA kits were used to quantify serum levels of insulin. glutathione (GSH), superoxide dismutase (SOD), and malondialdehyde (MDA) were measured using the homogenates of the pancreas in the light of instructions of reagent kits. Then, the homeostasis model assessment (HOMA) was used to evaluate IR, HOMA-IR = fasting insulin × fasting blood glucose/22.5, and as well as the function of β cell, HOMA-β = 20 × fasting insulin/(fasting blood glucose − 3.5 mmol/L).

### 2.5. Glucose-Stimulated Insulin Secretion (GSIS) Tests

INS-1 cells seeded in 24-well plates at 2 × 10^5^ cells per well, were pre-incubated for 30 min in KRB buffer (120 mmol/L NaCl, 4.8 mmol/L KCl, 2.5 mmol/L CaCl_2_, 1.2 mmol/L MgCl_2_, 24 mmol/L NaHCO_3_, and 1 g/L BSA) containing 2.8 mmol/L glucose and then incubated for 30 min in RPMI-1640 medium containing 2.8 mmol/L or 33.3 mmol/L glucose [25]. At the end of the incubation, the supernatant was collected for the measurement of insulin by radioimmunoprecipitation.

### 2.6. Pathological Study

Pancreatic tissues were fixed in 10% formalin solution for 48 h. Fixed tissues were processed for paraffin embedding and 5 μm sections were prepared for the histopathological, immunohistochemical, and morphometrical examination. Pancreatic sections were stained with hematoxylin and eosin (H&E) for routine histopathological examination as well as morphometric analysis at 200× magnification. Immunohistochemistry examination was used to detect ferritin light chain (FTL) and insulin protein content and distribution. Then, density data of the brown color of the immunostaining to be measured were then collected in an Excel sheet and submitted to statistical analysis. The sections were examined using a light microscope (Olympus) with an attached charge coupled device (CCD) digital camera (Mingmei Shot 60).

### 2.7. Transmission Electron Microscope

After animals were sacrificed, the trimmed pancreas tissue was fixed in glutaraldehyde (2.5%) and stored at 4 °C. The post-fixation was immobilized in 0.1 M osmium tetroxide prepared in 0.1 M phosphate buffer (pH 7.4) for 2 h at room temperature followed by dehydration, permeation, and embedding in Araldite. The tissues were polymerized at 60 °C for 48 h and cut into ultrathin sections (80–100 nm) using an ultramicrotome (Leica EM UC7, Solms, Germany). Subsequently, the thin slices on copper mesh grid were stained with uranyl acetate (2%) and lead citrate for 15 min at room temperature and observed under a transmission electron microscope (Tecnai G^2^ 20 TWIN, Hillsboro, OR, USA).

### 2.8. Western Blot Analysis

Pancreatic tissues were lysed in the radioimmunoprecipitation assay lysis buffer supplemented with 1 mM phenylmethanesulfonyl fluoride. The lysates were centrifuged and protein concentrations were measured by BCA protein assay kit (Beyotime Biotechnology, Shanghai, China). An equal amount of total protein was electrophoresed on a 12% acrylamide SDS gel and then electrotransferred to polyvinylidene fluoride membrane (Millipore Corp, Billerica, MA, USA). After being blocked with skimmed milk powder for 2 h at room temperature, the membranes were incubated overnight at 4 °C with the specific antibody for the target protein and 1 h with the corresponding secondary antibody. The membranes were washed and detected with EGL plus kit in a Western blotting detection system (Amersham Bioscience, Little Chalford, UK). The density of each target band was quantified by Image Pro-Plus 6.0 software (Media Cybernetics, Inc., Rockville, MD, USA) and normalized to β-actin as optical density.

### 2.9. mRNA Analysis

Total RNA was extracted and reverse transcribed, real-time RT-PCR was performed using the SYBR Green method and a 7900HT real-time PCR system (Applied Biosystems, Foster City, CA, USA). The value obtained for a specific gene product was normalized to the control gene β-actin.

### 2.10. Statistical Analysis

All animal data were expressed as mean ± SD, and cell experiment data were presented as mean ± SEM. For comparison of two groups, we used parametric Student’s t-test while its non-parametric alternative Mann–Whitney test if data did not follow a normal distribution. For three or more groups, we used a one-way analysis of variance (ANOVA); followed by Tukey’s post hoc test. All data were analyzed using the statistical program SPSS version 21 (SPSS INC., Chicago, IL, USA). The drawing was performed using Graph Pad Prism software version 5 (San Diego, CA, USA). A probability level of *p* < 0.05 was accepted as statistically significant.

### 2.11. ROS Determination

Frozen sections of the pancreas were stored at −20 °C, the ROS probes solution were diluted to the required concentration in PBS buffer and incubated at room temperature for 45 min. After the incubation, the tissues were washed with PBS 3 times, and excited with green light under a fluorescent microscope to observe and shoot red emission images of the cells, which represent ROS positive cells. For lipid ROS in the cell experiment, at the end of intervention, the cells were washed with PBS for 3 times, and then added with PBS containing C11-Bodipy^581/591^ (1 mmol/L) for 30 min in the oven at 37 °C. Then, the cells were washed with PBS for three times, and the red and green light were observed by fluorescence inverted microscope. The above operations should be done in the dark.2.12. cell culture INS-1.

INS-1 pancreatic cells were cultured in RPMI-1640 medium (containing 11.1 mmol/L glucose), supplemented with 10% fetal bovine serum (FBS), 1% penicillin and streptomycin, 50 μM 2-mercaptoethanol, 10 mM HEPES, 1 mM sodium pyruvate 100 U/mL penicillin, and 100 μg/mL streptomycin at 37 °C, in a humidified atmosphere containing 5% CO_2_.

## 3. Results

### 3.1. Induction of Diabetes and the Ameliorated Effect of Quercetin on the Changes of Biochemical Indexes

After 4-months feeding with HFD, the fasting blood glucose level of the high-fat feeding mice (HFD) group was 10.53 ± 0.43 mmol/L, which was 37% higher than that of the control mice 7.70 ± 0.60 mmol/L, indicating a well-established IR model by HFD regime (Supplemental Figure S1A, *p* < 0.001). The oral glucose olerance test (OGTT) showed that the blood glucose level in the high-fat group reached the highest level 45 min after the oral administration of 75 mg/kg body weight glucose solution and began to decrease, with a peak level of 24.08 ± 2.36 mmol/L, as compared to the findings of control mice reached peak level at 30 min after glucose intake, 15.30 ± 4.82 mmol/L (Supplemental Figure S1B, *p* < 0.05). The area under the blood glucose curve of the high-fat group was higher than that of the control group (Supplemental Figure S1C, *p* < 0.001). Following an insulin tolerance test with the intraabdominal injection of 0.75 IU/kg body weight insulin, the blood glucose level of the control mice decreased first and restored rapidly and maintained the lower level during almost 2 h of observation (Supplemental Figure S1D, *p* < 0.05).

**Figure 1:**
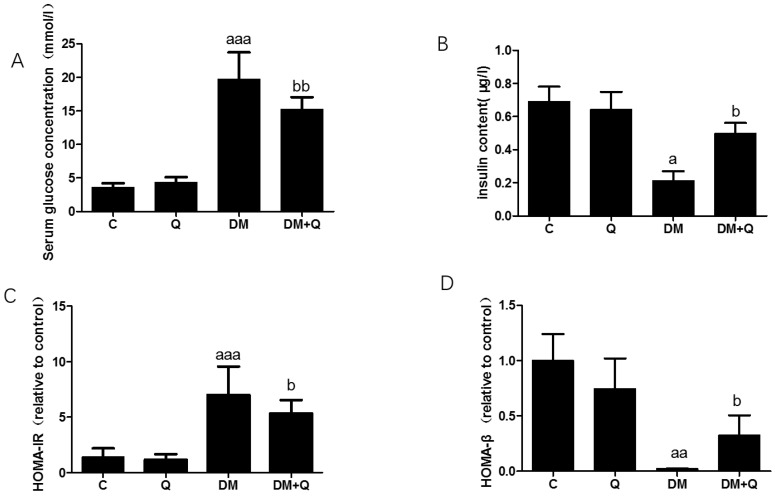
Induction of diabetes and measurement of pancreas function following high-fat feeding and streptozotocin injection and quercetin attenuates symptoms of T2DM in mice. (**A**). serum glucose concentration was measured by standard commercial assays kits. (mmol/L) (*n* = 10); (**B**). Insulin levels were determined by mouse-specific ELISA kits (*n* = 10); (μg/L); (**C**). The homeostasis model assessment of insulin resistance (HOMA-IR) = fasting insulin × fasting blood glucose/22.5 (*n* = 10); (**D**). The homeostasis model assessment of β cell function (HOMA-β) = 20 × fasting insulin/(fasting blood glucose − 3.5 mmol/L) (*n* = 10). Data were expressed as mean ± SD (a: *p* < 0.05 vs. C; aa: *p* < 0.01 vs. C; aaa: *p* < 0.001 vs. C; b: *p* < 0.05 vs. DM; bb: *p* < 0.01 vs. DM). T2DM, type 2 diabetes; DM, Diabetes mellitus; C, control group; Q, quercetin group.

To induce a typical T2DM model, low-dose of STZ was consecutively injected intraperitoneally for HFD-fed mice based on a well-established HFD-IR model. After the injection, the diabetic group showed typical symptoms of severe T2DM, including polydipsia, polyphagia, and weight loss (Table 1). Quercetin intervention reduced energy intake (DM group 15.1 ± 1.1 kcal/d vs. DM+Q group 13.3 ± 1.4 kcal/day, *p* < 0.01), water consumption (DM group 12.7 ± 0.6 g/day vs. DM+Q group 11.7 ± 0.3 g/day, *p* < 0.01), but did not restore the bodyweight of T2DM mice (26.52 ± 3.6 g vs. DM + Q group 26.59 ± 3.4 g, *p* > 0.05). After the mice were sacrificed, the serum insulin level was detected which was 70% lower than that in the control group, and quercetin intervention increased it to 2.3 times to DM group (Figure 1A, *p* < 0.05). While the blood glucose level was 5.5 times higher than that of the control group (DM group 19.76 ± 3.72 mmol/L vs. C group 3.56 ± 0.61 mmol/L, *p* < 0.001), quercetin intervention lowered blood glucose level, which was reduced to 15.22 ± 1.58 mmol/L (Figure 1B, *p* < 0.05). The calculated HOMA-IR index showed that the DM group had a significant increase compared with the control group (DM group 7.01 ± 2.33 vs. C group 1.42 ± 0.73, *p* < 0.05), and the insulin resistance index decreased to 5.35 ± 1.0 under the intervention of quercetin. (Figure 1C, *p* < 0.05). Further calculation of the HOMA-β index, DM group showed 23% of the normal group value and quercetin restored it to 37% (Figure 1D, *p* < 0.05).

### 3.2. The Protective Effect of Quercetin on Pathomorphological Changes of Islets T2DM Mice

**Figure 2:**
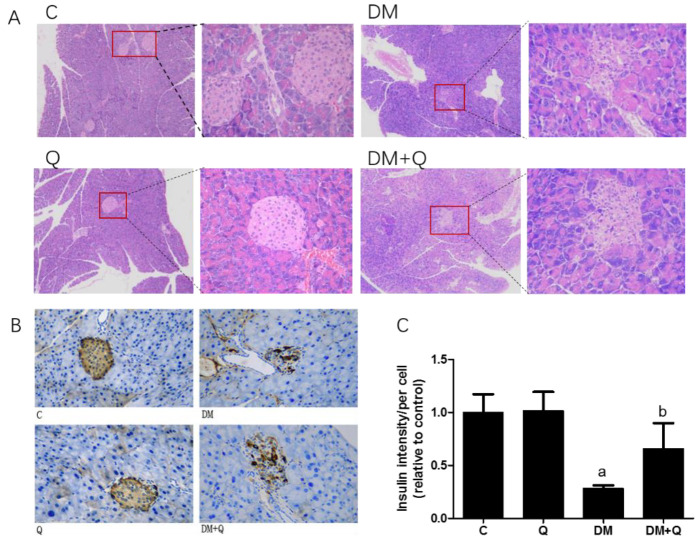
Morphological changes of islets in mice. (**A**). Fixed pancreatic tissue sections of mice stained with hematoxylin and eosin were observed by light microscope (image magnification: 400×) (*n* = 3); (**B**). Representative images of pancreatic sections immunohistochemically stained for insulin (dark brown) (image magnification: 400×) (*n* = 3); (**C**). Quantification of insulin level and distribution in islets were detected by immunohistochemically staining in pancreatic tissue (*n* = 3); Data were expressed as mean ± SD (a: *p* < 0.05 vs. C; b: *p* < 0.05 vs. DM).

HE staining showed that the pancreatic tissues of diabetic mice have dramatically depleted islets of Langerhans. The control islet architecture was complete, with clear boundary, and the β cell nucleus was round or ovoid, whereas the diabetic group islet structure damage was relatively serious, shrunken acellular and hypocellular islets were accompanied with nuclear karyorrhexis, focal vacuolar degeneration, and scattered inflammatory cell aggregated. Treatment with quercetin had displayed a markedly protective effect on the size and structure of the islet (Figure 2A).

**Figure 3:**
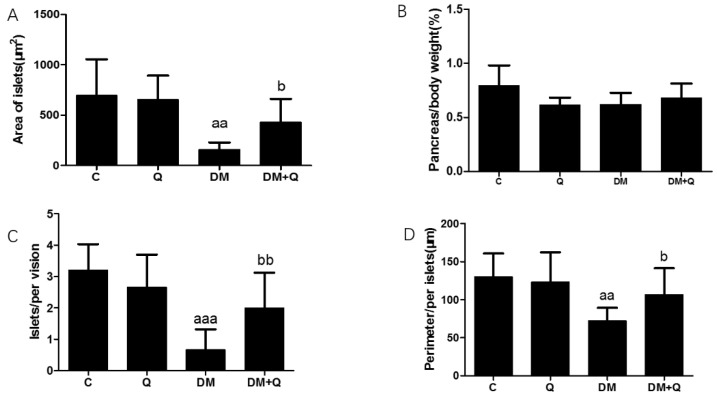
Morphological analysis of islets in mice. (**A**). Area of islets calculated by Mingmei camera at magnification: 200× (μm^2^) (*n* = 3); (**B**). Pancreas/body weight (%) (*n* = 10); (**C**). Numbers of islets/per vision calculated by Mingmei camera at magnification: 200× (*n* = 3); (**D**). Perimeter/per islets calculated by Mingmei camera at magnification: 200× (μm) (*n* = 3). Data were expressed as mean ± SD (aa: *p* < 0.01 vs. C; aaa: *p* < 0.001 vs. C; b: *p* < 0.05 vs. DM; bb: *p* < 0.05 vs. DM).

Further, the immunohistochemical experiment was carried out to observe the insulin level and distribution. Quercetin mitigated the disorganized islets and increased insulin antigen positivity cells in islets (Figure 2B), and the insulin optical density showed an 80% decrease in diabetic mice while quercetin restored the insulin content to 0.66-fold as compared to the control group (Figure 2C, *p* < 0.05). Morphometric results showed that the area, number, and perimeter of islets in the diabetic group has a significant reduction (0.23, 0.21, 0.55-fold, respectively, as compared with normal control, *p* < 0.05), and quercetin administration had a restorative effect which normalized the area of islets, the number of islets per vision and perimeter with 2.67, 3.03, 1.47-fold as compared to diabetic values. (Figure 3A,C,D *p* < 0.01). Although islets decreased in the diabetic group, there was no significant difference in the ratio of total pancreatic weight to body weight among the groups (Figure 3B *p* > 0.05).

### 3.3. Improvement of Quercetin on Iron Overload in the Pancreas of T2DM Mice

**Figure 4:**
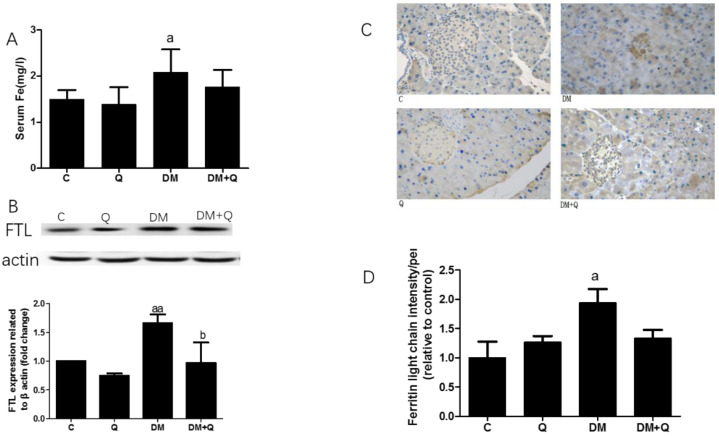
Effect of quercetin on iron levels in the pancreas of mice. (**A**). Serum iron level was measured by colorimetric kit according to the manufacturer’s instructions (mg/L) (*n* = 10); (**B**). Western blot analysis was performed to measure ferritin light chain (FTL) and quantified by Image-Pro Plus 6.0 software. Blotting with β-actin was used as a protein loading control (*n* = 3); (**C**). Representative images of pancreatic sections immunohistochemically stained for the ferritin light chain. (dark brown) (*n* = 3). (image magnification: 400×); (**D**). Quantification of the ferritin light chain level in islets detected by immunohistochemically staining in pancreatic tissue (*n* = 3). Data were expressed as mean ± SD. (a: *p* < 0.05 vs. C; aa: *p* < 0.01 vs. C; b: *p* < 0.05 vs. DM).

To further explore the cause of β cell death, the iron level was measured in serum and pancreatic tissues. As shown in Figure 4A, serum iron had a 1.38-fold increase as well as quercetin treatment restored the iron level (*p* < 0.05). Since serum iron cannot fully represent the iron level in pancreatic tissues, we detected the ferritin light chain by Western blotting, which increased 28% in the diabetic group, whereas quercetin lowered the level of ferritin (Figure 4B *p* < 0.01). To further confirm whether the site of iron deposition is islet or not, we used immunohistochemical staining to show where the ferritin is concentrated, and thereafter the result showed iron deposited in or near the center of islets, by an increase in the ferritin light chain of 1.94-fold as compared to the normal group, and quercetin treatment lowered the ferritin level by 35.7% (Figure 4C,D *p* < 0.05).

### 3.4. The Alleviated Effect of Quercetin on Lipid Peroxide and Antioxidant Ability with Changes of Protein Relating to Ferroptosis

**Figure 5:**
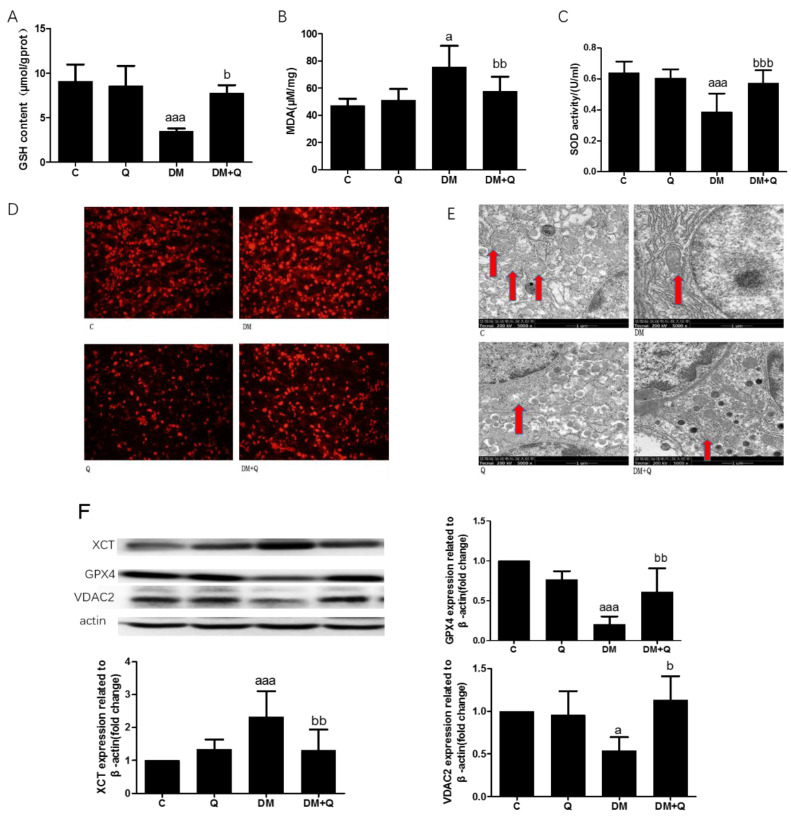
Effect of quercetin on improving the phenotype of ferroptosis. (**A**). Glutathione (GSH) content was measured using the homogenates of the pancreas in the light of instructions of reagent kits (*n* = 10); (**B**). Malondialdehyde(MDA) level was measured using the homogenates of the pancreas according to reagent kits (*n* = 10); (**C**). Superoxide dismutase (SOD) activity was measured using the homogenates of the pancreas according to the manufacturer’s instructions (*n* = 10); (**D**). Reactive oxygen species (ROS) level was determined by Dihydroethidium (DHE) probe; (**E**). Mitochondria (red arrows indicated) were observed from transmission electron microscope (TEM) images (5000×) showing representative mitochondria structure of β cells; (**F**). Western blot analysis was performed to measure xCT, GPX4, and VAC2 and quantified by Image-Pro Plus 6.0 software. Blotting with β-actin was used as a protein loading control. Data were expressed as mean ± SD (a: *p* < 0.05 vs. C; aaa: *p* < 0.001 vs. C; b: *p* < 0.05 vs. DM bb: *p* < 0.01 vs. DM; bbb: *p* < 0.001 vs. DM).

We identified the changes in pancreatic tissue antioxidant efficacy GSH, lipid peroxides (MDA), and SOD activity which are associated with ferroptosis. The level of GSH had a sharp decrease, 0.47-fold as compared to the normal group, and increased to 0.84-fold when quercetin is administrated (Figure 5A, *p* < 0.001). Lipid peroxide product MDA has increased 1.6 times while the treatment of quercetin exerts a protective effect on this lipid peroxide product (Figure 5B, *p* < 0.05). As well as another important antioxidant in the body, SOD exhibited the same trend, which reduced to 57% SOD activity (Figure 5C, *p* < 0.01), and restored to the normal level on the treatment of quercetin. We used the ROS fluorescence probe dihydroethidium (DHE) to detect the ROS level. As shown in Figure 5D, the diabetic group revealed a stronger red fluorescence than that of the control group, which is relieved significantly upon quercetin treatment. Moreover, in ferroptosis, what distinguishes it from other forms of death is its mitochondrial damage, which involves smaller mitochondria with increased membrane density (Maiorino et al., 2018). Ultrastructural observation of diabetic mice displayed reduced cell volume with immature secretory granules, fewer mitochondria, and endoplasmic reticulum is expanded to varying degrees in contrast with quercetin treatment which showed round or oval nuclei of the β cell, with a large number of mature secretory granules, medium density, active in synthesis insulin, thus resulting in high insulin levels. Further we found that in β cells of diabetic mice, mitochondria are significantly smaller, with shrunken and increased membrane density accompanied by progressive loss of cristae. (Figure 5E). To further investigate the mechanism of glucose toxicity-induced ferroptosis in β cell of T2DM mice, the ferroptosis-associated protein was detected by Western blotting, and the result showed that xCT increased 2.3-fold as compared to the normal group (Figure 5F, *p* < 0.001) and GPX4 is significantly reduced, accounting for 20% of control and quercetin normalized the proteins level (Figure 5F, *p* < 0.001). Another mitochondria membrane-associated protein VDAC2, which acts as an antioxidant role, is reduced significantly and is restored to normal by quercetin treatment (Figure 5F, *p* < 0.05). 

### 3.5. The Protective Effect of Quercetin on High Glucose Induced Ferroptosis in INS-1 Cells

**Figure 6:**
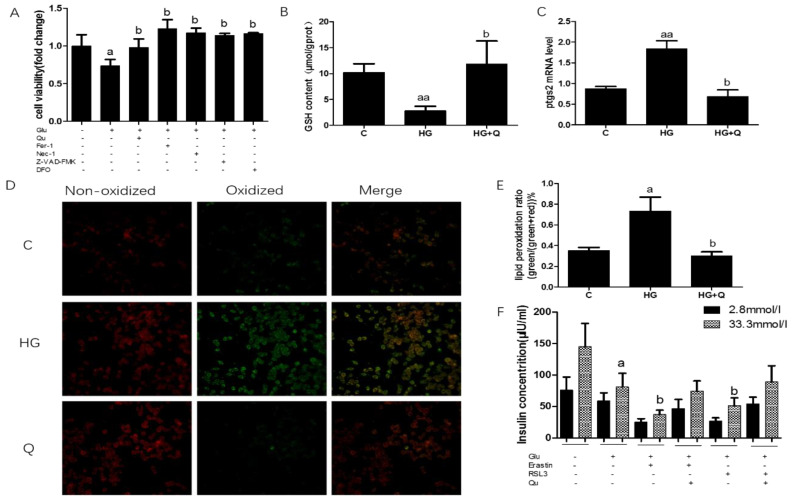
Quercetin alleviates ferroptosis-related phenotype in INS-1 cells induced by high glucose. (**A**). Cell viability was measured in INS-1 cells treated for 48 h with high glucose (33.3 mmol/L), quercetin (50 μM), Fer-1 (2 μM), Z-VAD-FMK (10 μM), Nec-1 (10 μM), deferoxamine (DFO) (25 μM); (**B**). GSH level was determined in INS-1 cells treated for 48 h with high glucose (33.3 mmol/L), quercetin (50 μM). (**C**). Ptgs2 mRNA level was determined in INS-1 cells treated for 48 h with high glucose (33.3 mmol/L), quercetin (50 μM), and normalized to β-actin mRNA; (**D**). Fluorescence micrographs of INS-1 labeled for 30 min with 1 μM C11-BODIPY^581/591^ (*n* = 3); (**E**). Quantification of fluorescence intensity calculated by dividing the green image by the sum of the green and red images (*n* = 3); (**F**). Glucose stimulated insulin secretion when treated for 48 h with erastin (1 μM) and RSL-3(1 μM). Data were expressed as mean ± SEM (a: *p* < 0.05 vs. C; aa: *p* < 0.01 vs. C; b: *p* < 0.05 vs. high glucose (HG)).

Previous reports found that high glucose causes several forms of cell death, including apoptosis and necrosis (Oh et al., 2019; Karatug and Bolkent 2018), and iron overload may play a role in this process, however, whether high glucose also causes ferroptosis is unknown, so we investigated this issue in the INS-1 cell line. Ferroptosis was assessed by cell viability, GSH content, ptgs2 mRNA, and lipid peroxidation. Cell viability decreased 27% when INS-1 cells were exposed to high glucose, and quercetin intervention restored the cell viability to normal (Figure 6A, *p* < 0.05). The specific ferroptosis inhibitors ferrostatin-1 (Fer-1) and deferoxamine (DFO) (an iron-chelating agent), reversed high-glucose-induced cell death while inhibitors of other forms of cell death, including Z-VAD-FMK (an apoptosis inhibitor) and necrostatin-1(a necroptosis inhibitor), also rescued cell viability induced by high glucose. Then, GSH content was measured, which decreased by 73% when cells were treated with high glucose, which can be reversed by quercetin treatment (Figure 6, *p* < 0.01). Next, we detected ptgs2 mRNA level, to find out that the ptgs2 mRNA level is 2.25 times than that of the control group, and quercetin lowered it to normal level (Figure 6C, *p* < 0.05).

Another characteristic of ferroptosis is the overproduction of lipid ROS, C11-bodipy was used to measure the amount of lipid ROS, fluorescent images showed that lipid ROS increased 2-fold when compared to normal control, while quercetin reversed this effect (Figure 6D,E, *p* < 0.05). To investigate the effect of ferroptosis on the responsiveness of INS-1 cells to glucose and ferroptosis agonist challenge. we next tested β cells’ function, namely insulin secretion ability in the INS-1 cell line, under high glucose, erastin, and RSL-3 treatment. The results were shown in Figure 5F. High glucose significantly decreased 45% of insulin secretion in INS-1 β cells, and agonists of ferroptosis, erastin, and RSL-3 aggravated insulin secretion to 17% and 18% of normal, respectively. This detrimental effect can be reversed by quercetin intervention but cannot be restored to the normal level (26% and 35%). (*p* < 0.05).

### 3.6. Figures and Tables

PBC death and dysfunction have been well-recognized as a pivot of the dramatically accelerated progression of T2DM. Quercetin displays beneficial antidiabetic qualities from several aspects [18]. Our data demonstrated that quercetin exerted a protective effect on PBC death concerning ferroptosis with iron chelators, GSH depletion, GPX4 inactivation, and lipid peroxidation. To our knowledge, it is the first time to identify novel molecular mechanisms of quercetin against T2DM by restraining pancreatic iron deposition and PBC ferroptosis, bringing a new insight into the pathophysiological role of pancreatic iron deposition and T2DM prevention by quercetin. These results give us an insight on T2DM, that ferroptosis was triggered due to the increased iron toxicity and lipid peroxidation, and therefore, it is intriguing to explore ferroptosis in PBC.

Our average quercetin dosage is 100 mg/kg·bw, which is validated in previous experiments by our team. Quercetin possesses great medicinal value, problems like low oral bioavailability and poor aqueous solubility make quercetin an unreliable candidate for therapeutic purposes. But diets are relatively simple in animal studies. About 93.3% of quercetin was metabolized in the gut, with only 3.1% metabolized in the liver after oral administration [26]. Therefore, the current design is aimed to explore the protective effect of quercetin on diabetes. Meanwhile, previous researchers suggested that quercetin was beneficial to control blood glucose in diabetic rats, which involved both C-peptide (proinsulin that is essential for mature insulin synthesis) secretion and stimulate insulin secretion [19,27]. The results in this study confirmed that high-fat diet and multiple-low dose STZ injection caused a significant increase in blood glucose level, as well as a sharp decrease in insulin level, which has been normalized by quercetin. Quercetin exerted a protective effect on pancreatic islets integrity, including the structure, area, and perimeter of pancreatic islets and increased insulin secretion function of PBC. The diabetic untreated group showed small, scattered, incomplete islets, and decreased insulin secretion, which was evident by histological observations and further immunohistochemical examination. Quercetin treatment showed increased β cells in islets and insulin optical density. Overall, these results indicate that the islets in diabetic conditions are severely damaged and thus caused decreased insulin secretion, suggesting that quercetin may improve β cell function by increasing the number of β cells and insulin secretion.

Iron dysfunction is associated with T2DM, especially iron deposition in islets. Excessive tissue iron may contribute to explain the loss of β cells in T2DM through upregulation of cellular iron import via DMT1 [28], which primes PBC to iron toxicity. Iron dysfunction also contributes to mitochondria defects, which is decisive for β cells in the progress of type 2 diabetes [29]. As a natural iron chelator, quercetin is reported to prevent liver injury in iron overload mice [30] (Zhang et al., 2011) and also demonstrated to effectively reduce iron deposition in endothelial cells [31]. In our study, T2DM mice exhibited increased iron concentration both in serum and pancreas tissues, and ferritin seemed to deposit particularly on or near islets manifested by immunohistochemical observation, showing a great iron overload in islets of pancreatic tissues and these iron depositions were attenuated by quercetin treatment. Taken together, these results indicated that iron deposited specifically on or near islets in T2DM mice, suggesting that iron overload involved in the progression of T2DM. Iron overload, also frequently seen in ferroptosis, maybe the de nexus linking ferroptosis and T2DM.

Ferroptosis is iron-dependent cell death, accumulating lipid peroxidation. GPX4 converts lipid hydroperoxides to lipid alcohols, and this process prevents the iron (Fe^2+^)-dependent formation of toxic lipid ROS [32]. Inhibition of GPX4 function leads to increased lipid ROS formation and lipid peroxidation, which can result in the induction of ferroptosis [33]. xCT (a specific light chain subunit of the cysteine/glutamate antiporter) transfers cystine into the cell for GSH synthesis to resist ferroptosis while VDAC2 is closely engaged in the steroidogenesis pathways and protection from oxidative stress [34]. In the current study, quercetin exerted a protective effect on ferroptosis with these protein levels. Western blotting showed that GPX4 is greatly reduced. A compensatory mechanism is activated to import cystine to synthesis more GSH to defense ferroptosis. These data suggested that ferroptosis participates in β cell death, leading to decreased insulin secretion and the ensuing events.

While ferroptosis contributes to pathological cell loss in many other diseases, the physiological function of ferroptosis in the PBC of T2DM is still elusive. In this study, we have presented compelling results implicating that ferroptosis might be an existing form of cell death that has not been discovered yet in β cell death. Various stimuli can trigger ferroptosis, such as lipid peroxidation and GSH depletion [35,36]. MDA is an end product of lipid oxidation, it is shown in our study that the MDA level increased in the diabetic group while quercetin lowered it to normal. Intracellular antioxidants GSH and SOD are essential in the protection of lipids from ROS. With large amounts of cytosolic ROS were detected, GSH content and SOD activity decreased significantly in diabetic mice while quercetin restored GSH and MDA levels. These results were consistent with the morphological changes of mitochondrial that specifically characterize ferroptosis, which displayed shrunken mitochondria with increased membrane densities manifested through TEM. Thus far, this research has shown that the results of increased lipid peroxide products, decreased antioxidant ability, and changes in mitochondrial morphology and structure support that ferroptosis participates in β cell death, leading to decreased insulin secretion and the ensuing events.

Further, this form of cell death in the INS-1 β cell line, and as we expected, high glucose stimulation increased lipid ROS in cells and the ptgs2 mRNA level, and at the same time decreased cell viability and GSH content, which is consistent with outcomes in animals. Furthermore, we used two classic agonists (erastin and RSL-3) of ferroptosis to induce cell death in the β cell line, and insulin secretion decreased significantly which proved that ferroptosis does exist in β cell death and influenced the secretion of insulin when treated with high glucose. Overall, these results confirmed that among all the forms of high glucose-induced β cell death, ferroptosis is a newly identified cell death that takes part in β cell dysfunction and death. Our results support that the protective effects of quercetin on mice pancreas partly occur by inhibiting ferroptosis. If the dosage can be more precisely controlled, we suggest more RCT trials to report on ferroptosis of human islets, which will provide more direct evidence on the prevention and treatment of T2DM.

## 4. Conclusions

In conclusion, these data identified ferroptosis as a novel form of cell death in PBC. Additionally, this study demonstrated the efficacy of quercetin role to achieve normal β cells’ function through amelioration of ferroptosis in T2DM. In addition, quercetin may represent potential therapeutic chemicals concerning ferroptosis in the development of T2DM.

## Figures and Tables

**Table 1:** Effect of quercetin on the changes of the body weight, energy, and water intake.

Group	Initial Weight (g)	Middle Weight (g)	Final Weight (g)	Energy Intake (kcal/day)	Water Intake (mL/day)
C	21.5 ± 1.1	27.9 ± 1.5	29.5 ± 1.9	13.7 ± 1.6	2.9 ± 0.5
Q	21.2 ± 0.8	27.5 ± 1.4	27.5 ± 1.5	13.5 ± 0.8	3.5 ± 0.9
DM	21.2 ± 1.2	33.4 ± 1.8 ^aaa^	26.5 ± 3.6 ^aaa^	15.1 ± 1.1 ^aaa^	12.7 ± 0.6 ^aaa^
DM + Q	21.1 ± 0.7	34.2 ± 2.9	26.6 ± 3.4	13.3 ± 1.4 ^bb^	11.7 ± 0.3 ^bb^

The male C57/6J mice were fed with the corresponding diet for 8 months. Data were collected weekly and expressed as mean ± SD (*n* = 10). Middle weight (g): Body weight before injection of streptozocin (STZ) (g): 4th month. ^aaa^: *p <* 0.001 vs. C; ^bb^: *p <* 0.01 vs. DM. 4. Discussion. DM, Diabetes mellitus; C, control group; Q, quercetin group

## Supplementary Materials

The following are available online at https://www.mdpi.com/2072-6643/12/10/2954/s1, Figure S1: Insulin resistance levels after 3 months high fat diet. (**A**). Fasting plasma glucose levels after 8 h mice fasted (*n* = 10); (**B**). Oral glucose tolerance test. (the dose used was calculated using the average body weight of the mice); (**C**). Quantification of the oral glucose tolerance test (OGTT) carried out using the area under the curve (AUC); (**D**). Insulin tolerance test. Data were expressed as mean ± SD (* *p* < 0.05; ** *p* < 0.01; *** *p* < 0.001 vs. C).
