# Supplementary material for: Quercetin Alleviates Ferroptosis of Pancreatic β Cells in Type 2 Diabetes
Source: Nutrients. 2020 Sep 27;12(10):2954. doi: 10.3390/nu12102954 (PMC7600916; doi:10.3390/nu12102954)
Supplement: Supplementary file 1 [file nutrients-12-02954-s001.pdf]

Supplementary Materials:

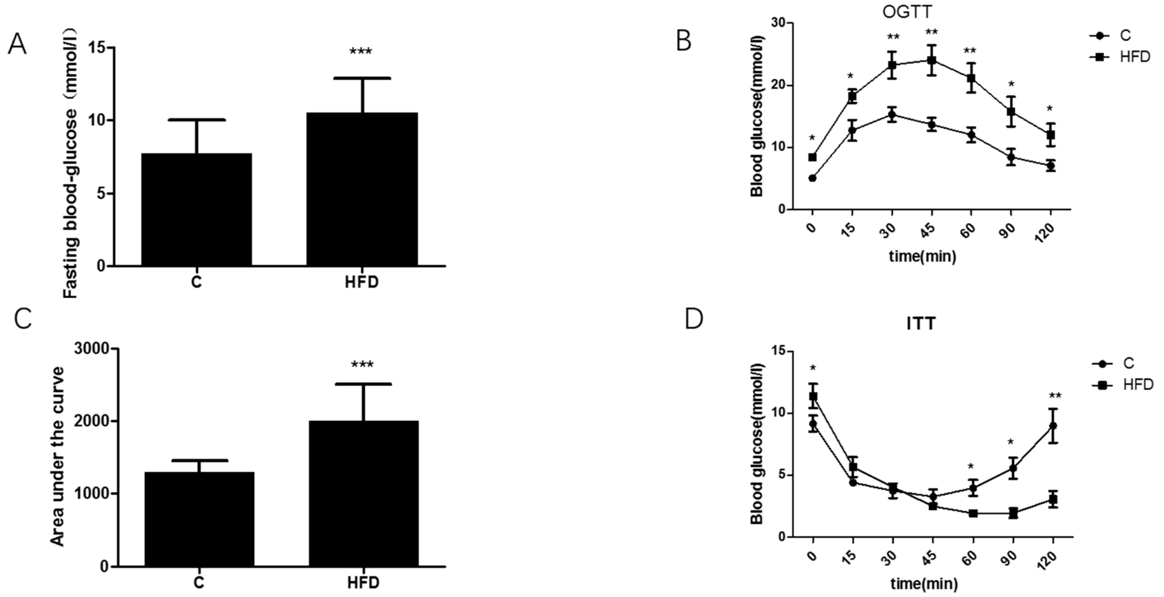

Supplymental Figure 1. Insulin resistance levels after 3 months high fat diet. A. Fasting plasma glucose levels after 8 hours mice fasted (n=10); B. Oral glucose tolerance test. (the dose used was calculated using the average body weight of the mice); C. Quantification of the oral glucose tolerance test (OGTT) carried out using the area under the curve (AUC); D. Insulin tolerance test. Data were expressed as mean ± SD (* $P < 0.05$; ** $P < 0.01$; *** $P < 0.001$ vs C).
